# Development and psychometric properties of the Compassion Fatigue Assessment Scale for Indian nurses

**DOI:** 10.1186/s12912-025-03670-0

**Published:** 2025-08-12

**Authors:** Shifali Sharma, Bharat Pareek, Harpreet Kaur, Priya Baby, Ramya Kundayi Ravi

**Affiliations:** 1 Saraswati Professional & Higher Education (SPHE) College of Nursing, Mohali, Punjab India; 2 Dr. Rajendra Prasad Govt. Medical College & Hospital, Kangra, Himachal Pradesh India; 3 Akal College of Nursing, Eternal University, Baru Sahib, Himachal Pradesh 173101 India; 4 MM College of Nursing, MM University Solan, Kumarhatti-Solan, Himachal Pradesh India; 5 College of Nursing, NIMHANS, Bangalore, India; 6 RAK College of Nursing, RAK Medical and Health Sciences University, Ras Al Khaimah, UAE

**Keywords:** Burnout, Compassion Fatigue, Factor analysis, Nurses, Scale, Secondary trauma

## Abstract

**Background:**

Compassion fatigue is a significant concern for nurses because they are constantly exposed to suffering. This study aimed to develop and validate a Compassion Fatigue Assessment Scale for Indian nurses.

**Methods:**

A quantitative, descriptive, and cross-sectional design was used to evaluate the psychometric properties, including the validity and reliability of the developed tool. A total of 200 nurses from two government and two private hospitals participated in the study between February and June 2022. Data were collected using a sociodemographic sheet, the Compassion Fatigue Assessment Scale, and the Professional Quality of Life Scale. Construct validity was assessed using principal component analysis, followed by examination of inter-domain correlations, while convergent validity was established using the Professional Quality of Life Scale. Reliability was tested using Cronbach’s α.

**Results:**

Principal component analysis with varimax rotation identified a ten-factor structure across the 36 items, explaining 60.34% of the variance. The extracted domains included “dissatisfaction and burnout,” “lack of emotions and sensitivity”, “lack of interest,” “secondary stress”, “hopelessness,” worthlessness, “overwhelming,” “lack of compassion”, “incompetency,” and “lack of productivity.” The overall Cronbach’s alpha was 0.88, with the subscale scores ranging from 0.70 to 0.91. The test–retest intra-class correlation coefficient was 0.93, indicating good stability.

**Conclusions:**

The Compassion Fatigue Assessment Scale demonstrated strong construct validity and internal consistency, making it a reliable tool for assessing and monitoring compassion fatigue among Indian nurses in research and professional settings.

**Supplementary Information:**

The online version contains supplementary material available at 10.1186/s12912-025-03670-0.

## Background

With organizations and administrators following a less traditional hierarchy or structure, workplaces, including hospitals, are becoming increasingly complex and difficult. In these evolving systems, the emotional demands of healthcare professionals are growing. Recognizing the urgent need to protect mental health in these dynamic work environments, the World Health Organization (WHO) and the International Labour Organization (ILO) jointly urge governments and organizations across public and private sectors to develop and implement strategies that prioritize mental health and well-being at work [1]. An often-overlooked but critical component of this, is compassion, both as a professional value and as a source of emotional strain. When consistently extended in high-stress environments without adequate support, compassion can lead to compassion fatigue among those in human service roles. According to ILO estimates, the mortality and disease burden caused by these unquantifiable risks, such as emotional exhaustion, stress, and burnout, are greater than those caused by measurable physical hazards [2].

Healthcare professionals, including doctors, nurses, and other frontline workers, operate in high-pressure environments characterized by intense workloads, emotional labor, and significant personal risk [3]. They have long been recognized as a particularly vulnerable group as they experience burnout, stress, and psychological distress [4–6]. Factors such as long working hours, staff shortages, insufficient protective equipment, changing clinical protocols, and the fear of transmitting infections to loved ones contribute to psychological distress [7–10]. This trend has been exacerbated only by the recent COVID-19 pandemic, which has placed considerable psychological strain on those working in the healthcare field. These outcomes not only compromise nurses’ well-being but also threaten the quality and safety of patient care, contributing to poorer patient outcomes and negatively impacting the overall performance of healthcare systems [5, 11].

Compassion fatigue, a pervasive issue in the healthcare industry, has emerged as a significant contributor to the alarming rates of burnout, psychological distress, and diminished physical and mental health among healthcare workers, particularly nurses [12–14]. There is growing evidence that nurses are disproportionately affected in this regard compared to other health professionals [15]. This could be because nurses are involved in direct care and are continually required to engage empathetically with individuals who are experiencing pain, distress, or suffering [13, 16] They are uniquely susceptible to compassion fatigue due to their continuous exposure to the emotional and traumatic experiences of their patients [17–19] and nurses often accumulate emotional stress, which over time can take a significant toll on their well-being and lead to a range of adverse outcomes [20].

Numerous detrimental physical, psychological, cognitive, and social effects have been linked to compassion fatigue, including decreased productivity, increased absenteeism, low job satisfaction, burnout, impaired professional judgment, and increased medical error. These effects ultimately result in unsafe and subpar patient care, poor patient outcomes, higher hospitalized patient mortality and morbidity, and an overall negative impact on healthcare organizations’ performance [21, 22]. Most healthcare workers enter the profession because they have passion to help others. Ironically, compassion fatigue has been identified as a “negative consequence and cost of caring that is capable of removing nurses’ compassionate caring forever” [23].

In Indian society, cultural expectations surrounding caregiving often place unrealistic demands on nurses for constant emotional availability and self-sacrifice, making compassion fatigue particularly pronounced and unique in their experience [24]. Even though it is imperative that administrators and healthcare organizations monitor compassion fatigue among nurses, there is no universally accepted and agreed upon definition of compassion fatigue. The scales commonly used globally to assess compassion fatigue may not accurately capture the nuances experienced by Indian nurses in their unique cultural and health care settings. Studies on Indian healthcare professionals have shown conflicting results and have highlighted cross-cultural variations when using these standard tools [25, 26]. This mismatch underscores the need for culturally tailored assessment instruments. Indian nurses face distinctive challenges, including resource constraints, hierarchical workplace dynamics, fluctuating patient loads, and limited psychosocial support, which have not been sufficiently addressed in existing scales developed in other countries. In this context, the investigators developed a new instrument to measure compassion fatigue among nurses in India.

## Aim of the study

This study aimed to develop and examine the psychometric properties of the Compassion Fatigue Assessment Scale (CFAS) in nurses.

## Methods

### Research design

This methodological study used a quantitative approach and a cross-sectional survey design.

The study was done in two phases.

#### Phase 1: conceptualization and item generation

The investigators conducted an extensive review of the literature available in major electronic databases to conceptually define the construct of compassion fatigue and examine existing measurement tools [18, 27–30]. To supplement the literature findings and capture real-world perspectives, semi-structured interviews were conducted with a purposive sample of 15 nurses selected through maximum variation sampling based on gender, years of experience, and levels of care delivery. The interviews lasted approximately 15–20 min and employed open-ended questions aimed at exploring nurses’ understanding and lived experiences of compassion fatigue within their clinical roles. For instance, participants were invited to reflect on their understanding of compassion in nursing, describe emotionally taxing care situations, and discuss challenges to maintaining compassionate care in high-pressure environments. All interviews were audio-recorded and were systematically reviewed to identify patterns that directly informed the item pool. Based on insights derived from the literature review and qualitative interviews, an initial pool of items was developed to reflect the dimensions of compassion fatigue among nurses [27, 31] These items were formulated as declarative statements representing the emotional, cognitive, and behavioral manifestations of compassion fatigue. The investigators reviewed the preliminary item pool to remove redundancies and to enhance content clarity. Each item was rated using a 4-point Likert-type scale indicating the frequency of experience: never [1], rarely [2], sometimes [3], and always [4].

#### Phase 2: psychometric evaluation

##### Face validity

The face Validity of CFAS was assessed by asking a group of ten nurses to evaluate the scale for clarity, importance, and comprehensiveness on a five-point Likert scale; they were also asked to identify any ambiguities in the statements [21, 32]. Later, they were asked to evaluate the importance of each item using a 5-point Likert-type scale (5 = *completely important* and I = *not important)*. An impact score greater than 1.5 was considered appropriate. Finally, the items were edited for language and grammar by investigators and experts.

##### Content validity assessment

The content validity of the instrument was ensured by calculating the Content Validity Ratio (CVR) and Content Validity Index (CVI). A panel of ten experts rated the relevance of each item using a three-point Likert scale. Items with a Content Validity Ratio (CVR) of less than 0.70 were excluded [21, 33, 34]. Four experts from the fields of nursing, instrument development, physician, and psychologist were invited to determine the relevance of the items using a three-point Likert scale. The item-level content validity index (I-CVI) and scale-level content validity index (S-CVI) were both calculated. Items with an I-CVI of 0.79 or more, 0.70–0.79, and less than 0.69 were respectively included, revised, and excluded, respectively. S-CVI values greater than 0.90 were considered acceptable [21, 35, 36].

##### Construct validity

Construct validity was assessed using principal component analysis (PCA) with varimax rotation to explore the underlying structure of the Compassion Fatigue Assessment Scale (CFAS). Sampling adequacy was evaluated using the Kaiser-Meyer-Olkin (KMO) test, and the suitability of the correlation matrix for factor analysis was examined using Bartlett’s test of sphericity. Factors were extracted based on eigenvalues greater than 1 and were supported by visual inspection of the scree plot. A minimum factor loading threshold of 0.40 was applied to retain items in the final factor solution [37]. Inter-domain correlations were calculated using Pearson’s correlation coefficients to examine the internal relationships among the identified components.

### Setting and sample

Data collection was done between February and June 2022. Convenience sampling was used to recruit participants. Participants were recruited from four hospitals in Punjab, a northwestern state in India, comprising two government and two private institutions. Of these, one was located in rural area and three in urban areas. All the hospitals selected for the study had a bed strength of more than 150. All nurses working in these hospitals, registered under the Punjab Nurses and Registration Council, aged ≥ 21 years, and working full-time in the selected hospitals in the past year were invited to participate in the study. Nurses who were ill in the past 3 months and who were not willing to participate were excluded. The sample size was estimated based on established methodological recommendations for scale development and validation, which suggest a participant-to-item ratio of at least 5:1–10:1 for factor analysis [38]. Given that the preliminary version of the Compassion Fatigue Assessment Scale (CFAS) included 38 items, a target sample size of 200 participants was deemed appropriate to ensure robust psychometric evaluation while accounting for potential nonresponse or incomplete data. All eligible nurses were invited to participate in this study. The data collection was terminated when the desired sample size was achieved.

#### Convergent validity

The convergent validity of the scale was determined by administering a compassion fatigue scale along with the previously validated “Professional Quality of Life” scale to nurses [37]. Pearson’s correlation analysis was performed to examine the correlation between the scores on the two scales.

#### Reliability assessment

The reliability of the scale was assessed using the internal consistency and temporal stability assessment techniques. The results of the internal consistency assessment are reported as Cronbach’s alpha. An alpha of greater than 0.70 was considered as acceptable internal consistency [39]. Moreover, stability assessment was performed using the test–retest technique, in which 30 nurses completed the CFAS twice with a 2-week interval. The correlation between the test and retest scores was examined using the intraclass correlation coefficient (ICC). ICCs greater than 0.75 were considered acceptable. Item analysis was performed to evaluate the relationship between individual items and overall scale. correlations between the items and the scale [40]. Items were retained if their item-total correlation exceeded 0.30 and their inter-item correlation remained below 0.70. Items falling outside these thresholds were considered for removal [40].

### Data collection

A paper based, self-administered questionnaire was used to collect data to test construct validity and reliability. Data collection was conducted in person at the participating hospitals, following the nurses’ clinical duty hours to avoid any disruption to routine patient care. Eligible nurses were approached in person at their respective hospital settings by the first author. This process was carried out under the supervision of the second and third authors, who also facilitated institutional coordination. The second and third authors obtained administrative permissions from the participating hospitals to ensure access and ethical compliance. The data collection process was carried out in a quiet space within the hospital premises, and each participant took approximately 30 min to complete the questionnaire.

**Fig. 1:**
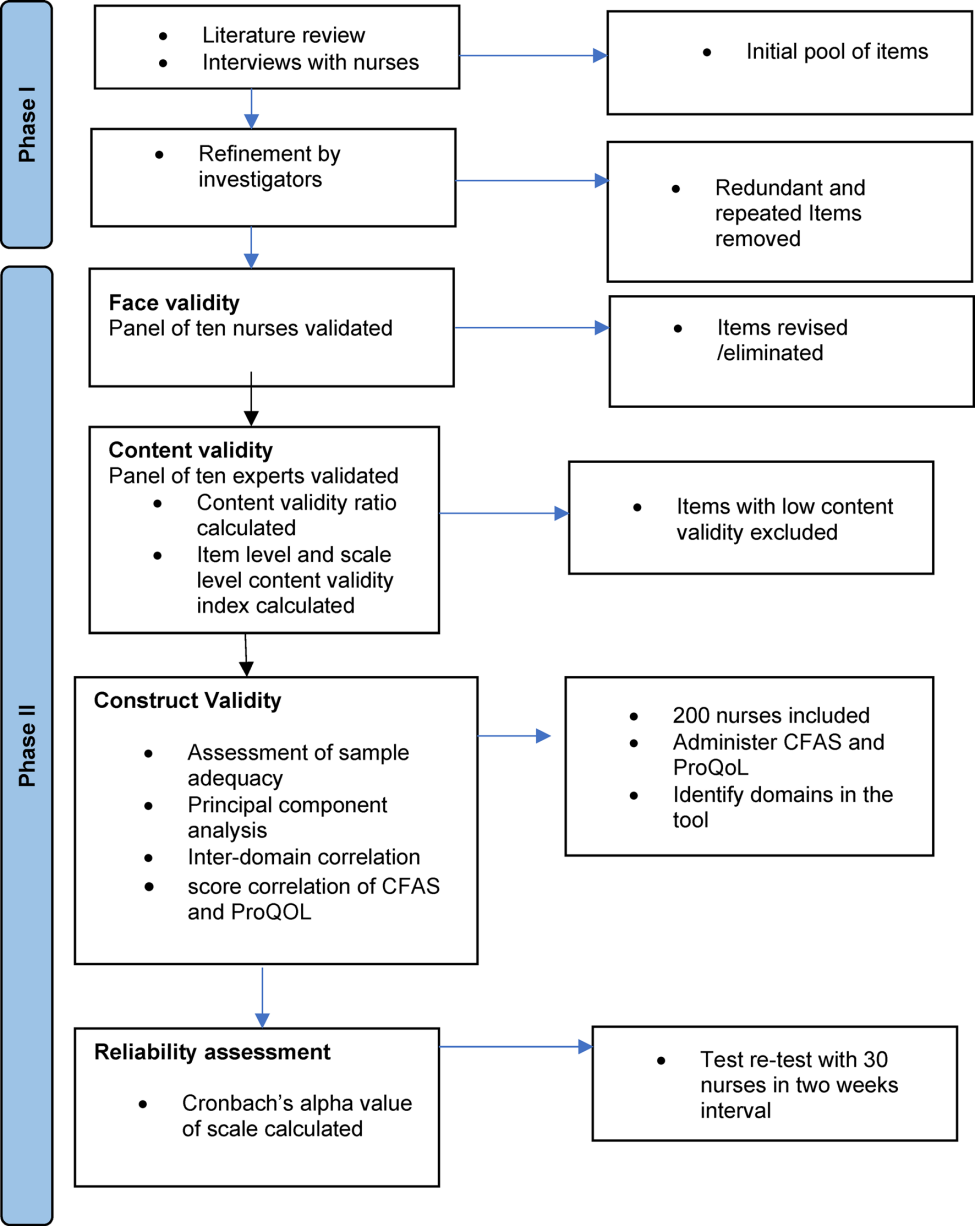
Flow chart of the tool development of CFAS

The questionnaire was administered in English, as all participating nurses were trained in English and used it as their primary language of communication in academic settings. Therefore, translation or linguistic validation procedures are not required. The first section of the questionnaire contained six sociodemographic items that were used to gather information regarding age. The second part contained the newly developed 36 item CFAS. The third section included the validated “professional QOL [37]. The Professional Quality of Life Scale (ProQOL)], is an open access instrument which is freely available for academic and research use. The tool development process is described in Fig. 1.

### Ethical considerations

The study protocol was reviewed and approved by the Institutional Ethics Committee of Saraswati Professional & Higher Education (SPHE) College of Nursing (Approval No. IEC/SPHE/N2021/78, dated 06.12.2021). Following this, administrative permission was obtained from the selected hospitals by the second and third authors. Eligible nurses were then approached in person at their respective hospital settings by the first author. The purpose of the study was explained, and written informed consent was obtained prior to participation. Data collection was conducted after clinical duty hours to ensure minimal disruption to patient care. Privacy of information, anonymity, and confidentiality of data were assured to the participants before data collection. They were informed of the voluntary nature of the study and given the right to withdraw at any time they wished to.

### Statistical analysis

The Statistical Package for the Social Sciences (SPSS) version 24 was used to analyze the data acquired from the participants. Frequencies, percentages, means, and standard deviations were used to describe variables. A p value < 0.05 was used to establish statistical significance.

## Results

### Phase 1

The pool initially contained 83 items. At the end of Phase One, 42 items were retained following the literature review, information elicited from nurses during interviews, and refinement by investigators to ensure clarity and relevance.

#### Phase 2: psychometric analysis

##### Face validity

During the face validity assessment, six items were revised to enhance the conceptual and linguistic clarity. Of the items assessed, seven obtained an impact score below the threshold of 1.5. Based on the investigator’s consensus and subsequent refinement, four of these items were eliminated, while three were retained following appropriate modifications. The resulting scale comprised 38 items that were further reviewed and refined by a panel of experts to ensure grammatical accuracy and terminological precision. The panel comprised of the study investigators, as well as an English language editor and a professional linguist with expertise in academic and healthcare communication. The language experts held postgraduate qualifications in English literature and linguistics, with experience in editing scientific writing.

##### Content validity

In this step, two items with a CVR of less than 0.78 were excluded. Two items with an I-CVI of 0.70–0.79 were revised. The S-CVI was 0.90. At the end of this step, the CFAS contains 36 items.

##### Construct validity

Of the four hospitals selected for the study, 274 nurses who met the inclusion criteria were invited to participate. Among them, 58 nurses declined to participate, primarily citing time constraints. Consequently, questionnaires were distributed to 216 nurses, of whom 200 returned fully completed questionnaires, yielding a response rate of 92.8%. The remaining 16 nurses did not return the questionnaire, attributing their nonresponse to lack of time, inability to complete the questionnaire after initial engagement, and other personal reasons. Most participants were females (81%) and married (51%). Detailed characteristics of the nurses who completed the survey are shown in Table 1.

**Table 1:** Characteristics of participants

Variables	f	%
**Age**		
a) 20–25 years	74	37
b)26–30 years	58	17
c) 31–35 years	34	29
d) > 35 years	34	17
**Gender**		
a) Male	38	19
d) Female	162	81
**Marital status**		
a) Married	102	51.0
b) Unmarried	97	48.5
**Professional qualification**		
a) ANM	10	5.00
b) GNM	93	46.50
c) B.Sc (Nursing)	89	44.50
d) M.Sc (Nursing).	8	4.00
**Experience**		
a) 6 months- 1 year.	24	12.0
b) 1–2 years.	47	15.5
c) 2–3 years.	31	23.5
d) > 3 years	98	49.0
**Hospital**		
a) Private.	98	49
b) Government	102	51
**CNE/Workshop/conference in last 1 year**		
a) Yes	84	42
b) No	116	58
**Subscription of nursing journal**		
a) Yes	26	13
b) No	174	87
**Any advance course in nursing skills**		
a) Yes	48	24
b) No	152	76
**Membership of professional organization**		
a) Yes	52	26
b) No	148	74

### Assessment of sample adequacy and sphericity of compassion fatigue scale

Using the Kaiser-Meyer-Olkin (KMO) test to assess sampling adequacy, a result of 0.84 was obtained. This exceeds the recommended minimum of 0.60, indicating meritorious sampling adequacy according to Kaiser’s (1974) classification [41]. This suggests that the sample size (*N* = 200) and pattern of correlations among the variables were sufficient for conducting principal component analysis. Bartlett’s Test of Sphericity yielded a *p*-value of < 0.001, indicating that the correlation matrix significantly differed from an identity matrix. This confirms that the variables are suitably interrelated, meeting the assumptions necessary to proceed with PCA for dimensionality reduction and structure exploration. (Table 2).

**Table 2:** Measure of sampling adequacy *N* = 200

Kaiser-Meyer-Olkin measure of sampling Adequacy		0.843
Barlett’s Test of Sphericity	Approx. Chi Square	2.302
	Degree of freedom (df)	630
	**Sig**	0.000

The extraction method was used to determine the total variance in the scale items. The principal component resulted in the extraction of ten factors with eigenvalues > 1, and an examination of the scree plot also suggested the same ten primary factors. These ten factors accounted for 60.33% of the variance (Table 3).

**Table 3:** Factor extraction summary using principal component analysis

Domains	Initial Eigen values			Extraction Sums of Squared Loadings			Rotation Sums of Squared Loadings		
	Total	% of variance	Cumulative %	Total	% of variance	Cumulative %	Total	% of variance	Cumulative
1. 2. 3. 4. 5. 6. 7. 8. 9. 10.	8.22 2.49 1.90 1.75 1.48 1.33 1.31 1.15 1.07 1.04	22.85 6.92 5.28 4.86 4.13 3.71 3.65 3.21 2.99 2.90	22.85 29.77 35.05 39.92 44.05 47.76 51.41 54.63 57.63 60.53	8.22 2.49 1.90 1.75 1.48 1.33 1.31 1.15 1.07 1.04	22.85 6.92 5.28 4.86 4.13 3.71 3.65 3.21 2.99 2.90	22.85 29.77 35.05 39.92 44.05 47.76 51.41 54.63 57.63 60.53	3.28 2.75 2.63 2.37 2.20 2.07 2.03 1.68 1.42 1.31	9.12 7.66 7.31 6.60 6.12 5.75 5.64 4.69 3.96 3.64	9.12 16.79 24.10 30.71 36.83 42.59 48.23 52.92 56.88 60.53

#### Convergent validity

A high convergent validity (*r* = 0.82) was determined using the score correlation of both CFAS and ProQOL.

#### Reliability assessment

Cronbach’s alpha value of the CFAS was 0.88, indicating excellent internal consistency. Cronbach’s alpha values of the ten extracted factors were 0.82, 0.70, 0.76, 0.75, 0.77, 0.71, 0.7. 0.79. 0.88 and 0.90, respectively. The test–retest reliability, assessed using the intraclass correlation coefficient (ICC), was 0.93, demonstrating excellent temporal stability. The standard error of the measurement was 7.8, indicating an acceptable level of precision for the individual score estimates. Table 4 presents the results.

Domain I had six items linked to ‘job satisfaction and burnout’, with an individual variance of 22.85, factor loading range from 0.72 to 0.45, and domain reliability of.82. Domain II contained five items on ‘lack of emotions and sensitivity’ with a total variance of 6.92, a factor loading range from 0.64 to 0.52, and a domain reliability of 0.70. Domain III contained six items on ‘lack of interest’ with a total variance of 5.28, a factor loading range from 0.66 to 0.48, and a domain reliability of.76. Domain IV contained four items on ‘Secondary stress’ with a total variance of 4.86, factor loading range from 0.75 to 0.45, and domain reliability of.764. Domain V had four items pertaining to ‘hopelessness’, with a total variance of 4.13, factor loading range of 0.64 to 0.42, and domain reliability of 0.77. Domain VI contained three items associated with ‘Worthlessness’, with a total variance of 3.71, factor loading range of 0.79 to 0.48, and domain reliability of 0.710. Domain VII contained three items on ‘Overwhelming’, with a total variance of 3.65, factor loading range of 0.76 to 0.48, and domain reliability of 0.69. Domain VIII contained three ‘lack of compassion’ items with a total variance of 3.21, factor loading range of 0.66 to 0.42, and domain reliability of 0.79. Domains IX (Incompetency) and X (Lack of productivity) each featured one item, factor loading range of 0.71 to 0.66 with a total variance of 2.99 and 2.90.

**Table 4:** Factor loading, variance, and cronbach’s alpha based on principal component analysis *N*=200

Items	Factor Loading	Total variance explained	Cronbach’s Alpha
**Domain I- Dissatisfaction and burnout (Total no of items = 6)**			
I am dissatisfied with my job.	0.72	22.85	0.82
I am dissatisfied with the nature of my work.	0.69		
I feel tired while working.	0.64		
I am unhappy with my work.	0.57		
I feel that I have become very arrogant and harsh towards people since I took this job.	0.52		
I feel that there is no one to talk about highly stressful experiences.	0.45		
**Domain II- Lack of emotions and sensitivity (Total no of items = 5)**			
I fail to perceive patient’s situation and feelings.	0.64	6.92	0.705
I do not take extra mile to help the patient.	0.62		
I feel disconnected to my patients and co-workers.	0.53		
I lack curiosity in learning new things.	0.52		
I do not communicate my understandings to the client.	0.52		
**Domain III- Lack of interest. (Total no of items = 6)**			
I do not feel interested while handling new cases.	0.65	5.28	0.764
I avoid spending time with patients.	0.59		
I feel boring while working with some people I helped.	0.56		
I feel depressed of caring critically ill patients on regular routine	0.52		
I feel exhausted when I wake up in the morning and have to face another day at work	0.50		
I think that I am experiencing a trauma of a person I care for	0.48		
**Domain IV- Secondary stress (Total no of items = 4)**			
I feel pre-occupied with the person I helped.	0.75	4.86	0.756
I feel like being in danger while caring traumatized patients	0.56		
I get intrusive thoughts of the terrible experiences of caring others.	0.52		
I feel that I am not successful in my role as a helper or caregiver	0.45		
**Domain V- Hopelessness (Total no of items = 4)**			
I feel that I am not succeeding as a helper or caregiver.	0.64	4.13	0.770
I feel very weak as a result of my job as a helper	0.56		
I do not view my failings as part of the human condition.	0.54		
I do not seem to recover easily after facing daily troubling events	0.42		
**Domain VI- Worthlessness (Total no of items = 3)**			
I have a sense of worthlessness associated with my work.	0.79	3.71	0.710
I really do not care about my patient.	0.59		
I do not feel sincere while caring patients.	0.48		
**Domain VII- Overwhelming (Total no of items = 3)**			
I feel overwhelmed while caring for critically ill patients.	0.76	3.65	0.7
My patient’s stress affects me deeply.	0.56		
I experienced frightening dreams at night of those I helped in a day.	0.48		
**Domain VIII- Lack of compassion (No of items = 3)**			
I feel drained rather than energized after caring for others	0.66	3.21	0.792
I do not feel proud of what I do to help others	0.57		
I do not feel a sense of compassion in the work I do	0.43		
**Domain IX- Incompetency(No of items = 1)**			
I find myself incompetent in my work	0.71	2.99	0.88
**Domain X- Lack of productivity (No of items = 1)**			
My productivity at work has been reduced	0.66	2.90	0.91
Total (10 Domains and 36 Items)	0.79 to 0.42	60.53	0.88

### Inter domain correlation

Inter-domain correlations were examined to assess the relationships between the ten identified components. As seen in Table 5, moderate to strong positive correlations were found among most domains, thus supporting the internal coherence of the construct.

**Table 5:** -Inter domain correlation matrix of compassion fatigue assessment Scale:-

Domains	Dissatisfaction and Burnout	Lack of Emotions and Sensitivity	Lack of Interest	Secondary stress	Hopelessness	Worthlessness	Overwhelming	Lack of compassion	Incompetency	Lack of productivity
Dissatisfaction and Burnout	1	0.475^**^	0.497^**^	0.329^**^	0.519^**^	0.299^**^	0.355^**^	0.048	0.032	0.295^**^
Lack of Emotions and Sensitivity	0.475^**^	1	0.580^**^	0.432^**^	0.555^**^	0.380^**^	0.375^**^	0.035	0.034	0.325^**^
Lack of Interest.	0.497^**^	0.580^**^	1	0.542^**^	0.599^**^	0.280^**^	0.458^**^	0.043	− 0.042	0.233^**^
Secondary stress	0.329^**^	0.432^**^	0.542^**^	1	0.395^**^	0.129	0.505^**^	− 0.017	0.029	0.110
Hopelessness	0.519^**^	0.555^**^	0.599^**^	0.395^**^	1	0.304^**^	0.400^**^	− 0.028	− 0.006	0.320^**^
Worthlessness	0.299^**^	0.380^**^	0.280^**^	0.129	0.304^**^	1	0.101	− 0.017	0.165	0.391^**^
Overwhelming	0.355^**^	0.375^**^	0.458^**^	0.505^**^	0.400^**^	0.101	1	− 0.064	− 0.025	0.106
Lack of compassion	0.048	0.035	0.043	− 0.017	− 0.028	− 0.017	− 0.064	1	− 0.009	0.035
Incompetency	0.032	0.034	− 0.042	0.029	− 0.006	0.165*	− 0.025	− 0.009	1	0.095
Lack of productivity	0.295**	0.325**	0.233**	0.110	0.320**	0.391**	0.106	0.035	0.095	1

**Correlation is significant at the level 0.01 level (2-tailed)

*Correlation is significant at the 0.05 level (2-tailed)

### Cut-off score determination and ROC analysis

**Fig. 2:**
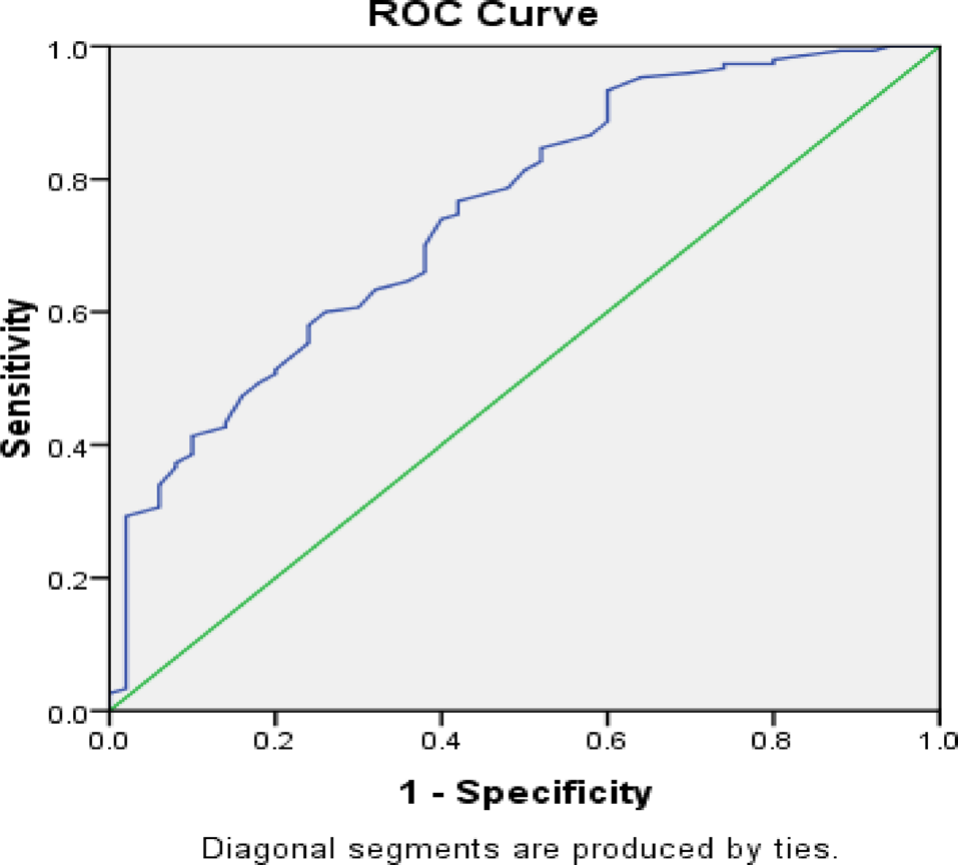
Receiver operating curve

To determine an optimal cut-off score for identifying individuals at risk of compassion fatigue, Receiver Operating Characteristic (ROC) curve analysis was conducted. The ROC curve was used to evaluate the sensitivity (true positive rate) and specificity (true negative rate) of the Compassion Fatigue Assessment Scale (CFAS) in classifying participants based on their responses. The range of the scale is from 36 to 144. The analysis identified 71.5 as the best cut-off score, with a sensitivity of 0.70 and a specificity of 0.38 (Fig. 2). The cutoff was selected to maximize sensitivity in identifying individuals experiencing symptoms of compassion fatigue, given the scale’s potential use as a screening tool.

## Discussion

This study developed and validated a new Compassion Fatigue Assessment Scale (Appendix 1) for Indian nurses. The results revealed that the newly developed scale with ten factors and 36 - items has strong psychometric properties, including good construct validity and internal consistency (Cronbach’s α = 0. 88), and test-retest reliability (ICC = 0.93), making it a valid and reliable tool for assessing and monitoring compassion fatigue among nurses. The scale identified 10 domains associated with compassion fatigue: These ten factors “dissatisfaction and burnout,” “lack of emotions and sensitivity,” “lack of interest,” “secondary stress,” “hopelessness,” “worthlessness,” “overwhelming,” “lack of compassion,” “incompetency” and “lack of productivity” accounted for 60.33% of the variance.

The multidimensional structure of compassion fatigue observed in this study corroborates previous studies and the existing compassion fatigue scales. The Compassion Fatigue Self-Test (CFST) was one of the first measures developed based on clinical experience to assess both compassion fatigue and job burnout. The scale has 40 items and was further developed to measure compassion satisfaction with the addition of 16 items [42, 43]. Continued work on the scale has resulted in the development of a Professional Quality of Life Scale. The Professional Quality of Life Scale with three subscales namely “compassion satisfaction,” “burnout,” and “secondary traumatic stress” is one of the most widely used tools globally to measure compassion fatigue among nurses [37, 44] Another common tool, the Burnout Measure is a 21 - items self-report measure that assesses the level of an individual’s physical, emotional, and mental exhaustion. Although the tool is useful for assessing general burnout, it does not address other critical aspects of compassion fatigue [45]. The newly developed CFAS has 10 domains, and has been demonstrated to have excellent construct and cross-validation, which is in line with these compassion fatigue scales. This scale was tailored to the unique stressors experienced by Indian nurses working in resource limited settings. Furthermore, the culture of self- sacrifice, the hierarchical nature of healthcare systems, demanding work schedules, and resource constraints specific to India might have influenced identification of the factors such as hopelessness and worthlessness [46].

Of all the domains, ‘Dissatisfaction and Burnout’ emerged as the strongest, with a variance of 22.85% and Cronbach’s α = 0.82. This finding aligns with previous research that highlights burnout as a significant contributor to compassion fatigue [46]. The domain “Lack of Emotions and Sensitivity”, with a variance of 6.92% (α = 0.705), reflects the emotional detachment nurses may develop due to prolonged exposure to challenging situations. Similar findings were noted in earlier studies in which emotional disconnection was reported as a key symptom of compassion fatigue and a mechanism adopted by nurses to work in high-stress environments [29, 47, 48]. The ‘lack of interest’ domain accounted for 5.28% of the variance (α = 0.764), suggesting apathy and reduced motivation of nurses in professional duties. This is consistent with earlier studies that have identified emotional numbing as a coping mechanism against traumatic exposure [49]. Secondary stress (variance = 4.86%, α = 0.756), a well-documented phenomenon in the caregiving profession, has also emerged as a domain in CFAS. Long duty hours, heavy workload, and lack of support are presumed to be some of the drivers of secondary traumatic stress among Indian nurses, as they work in resource-scarce settings [19]. Hopelessness and worthlessness have emerged as other significant domains highlighting how compassion fatigue erodes self-esteem and professional identity [50]. The identification of “overwhelming” as a domain corroborates earlier literature suggesting that persistent occupational stress, particularly within critical care contexts, is significantly associated with impaired mental health outcomes among professionals [29]. A sense of incompetency and lack of productivity were also found among study participants who doubted their professional abilities [13, 50].

The present study followed established best practices for scale development, as recommended by experts in psychometric research [51, 52]. The Compassion Fatigue Assessment Scale (CFAS) demonstrates robust psychometric properties, positioning it as a reliable and valid instrument for assessing compassion fatigue among Indian nurses. Its internal consistency and temporal stability support its use in both research and clinical settings. The scale aligns well with existing measures in the field, further reinforcing its construct validity and practical relevance [29, 53].

### Strengths, limitations and recommendations

The scale demonstrated excellent psychometric properties, including high internal consistency (Cronbach’s α = 0.88) and test-retest reliability (ICC = 0.93), indicating that the CFAS was reliable and stable over time. The identification of ten specific domains of compassion fatigue, some of which address the unique cultural and occupational stressors faced by Indian nurses, is another notable strength of this study. Although the researcher conducted extensive statistical analyses to build the scale, some constraints were unavoidable during the course of the study. The use of a convenience sampling method may have introduced a selection bias, limiting the representativeness of the sample to nurses in other healthcare settings or regions. Discriminant validity was not assessed in the present study, which may limit the completeness of the construct validation. Another limitation is the lack of linguistic validation of the scale for use in non-English-speaking or multilingual populations, which may limit its applicability to the diverse linguistic groups in India. Future research should include measures of unrelated constructs to evaluate discriminant validity and further strengthen the psychometric profile of CFAS. Future studies are also recommended to evaluate the predictive validity of the CFAS by examining its ability to forecast clinically and professionally relevant outcomes such as nurse burnout, job attrition, absenteeism, and quality of patient care. Longitudinal designs that assess baseline CFAS scores and follow-up at defined intervals can provide valuable insights into the utility of this scale in predicting risk over time. Advanced psychometric techniques such as Confirmatory Factor Analysis (CFA) and Item Response Theory (IRT) were not applied in this initial study but are recommended for future validation efforts to further examine model fit and item-level functioning.

## Conclusions

The Compassion Fatigue Assessment Scale (CFAS) is a psychometrically robust, culturally relevant, and practically useful tool for assessing compassion fatigue among nurses in the Indian context. Its demonstrated reliability and validity make it suitable for identifying at-risk individuals, enabling timely and targeted interventions to strengthen resilience, reduce burnout, and promote overall wellbeing. By integrating CFAS into routine organizational assessments, healthcare institutions, especially those operating in resource-limited settings, can proactively monitor and support the emotional health of their nursing workforce. This scale can also serve as an outcome measure for evaluating the effectiveness of interventions aimed at reducing compassion fatigue, thereby contributing to evidence-based practice. Future research should aim to validate the CFAS across diverse populations and healthcare environments in India, enhancing its generalizability. Additionally, exploring its predictive validity in relation to critical outcomes, such as staff turnover, job satisfaction, and quality of patient care, will be valuable.

## Supplementary Information

Below is the link to the electronic supplementary material.

Supplementary Material 1

## Data Availability

The datasets generated and/or analysed during the current study are available within the manuscript.

## Abbreviations

CFAS: Compassion Fatigue Assessment Scale
PCA: Principal Component Analysis
ProQOL: Professional Quality of Life
ILO: International Labor Organization
WHO: World Health Organization
CVR: Content Validity Ratio
CVI: Content validity index
I-CVI: Item-level content validity index
S-CVI: Scale-Level Content Validity Index
SPSS: Statistical Package for Social Sciences
ROC: Receiver Operating Characteristic
ICC: Intraclass Correlation coefficient
KMO: Kaiser-Meyer-Olkin
CFST: Compassion fatigue self-test
IRT: Item response theory
