# Supplementary material for: Development and psychometric properties of the Compassion Fatigue Assessment Scale for Indian nurses
Source: BMC Nurs. 2025 Aug 12;24:1061. doi: 10.1186/s12912-025-03670-0 (PMC12344833; doi:10.1186/s12912-025-03670-0)
Supplement: Supplementary file 1 — Supplementary Material 1 [file 12912_2025_3670_MOESM1_ESM.docx]

**Annexure 1**

**COMPASSION FATIGUE ASSESSMENT SCALE FOR NURSES**

**INSTRUCTIONS:-**

1. This scale consists of 36 items/ statements on compassion fatigue. Consider each of the following statement about you and your current working /life situation. Please rate honestly by selecting appropriate response from 1 to 4 which best reflect you to experience following characteristics at your working area in last 3 months.
2. Your responses will be confidential.
3. The more honestly you answer the more valuable the results will be.

| **Domain I- Dissatisfaction and Burnout** |
| --- |

| 1. I am dissatisfied with my job. | | | |
| --- | --- | --- | --- |
| Never | Rarely | Sometimes | Always |
| 1 | 2 | 3 | 4 |

| 1. I am dissatisfied with the nature of my work. | | | |
| --- | --- | --- | --- |
| Never | Rarely | Sometimes | Always |
| 1 | 2 | 3 | 4 |

| 1. I feel tired while on working. | | | |
| --- | --- | --- | --- |
| Never | Rarely | Sometimes | Always |
| 1 | 2 | 3 | 4 |

| 1. I am unhappy with my work. | | | |
| --- | --- | --- | --- |
| Never | Rarely | Sometimes | Always |
| 1 | 2 | 3 | 4 |

| 1. I feel that I become very arrogant and harsh towards people since I took this job. | | | |
| --- | --- | --- | --- |
| Never | Rarely | Sometimes | Always |
| 1 | 2 | 3 | 4 |

| 1. I feel that there is no one to talk about highly stressful experiences. | | | |
| --- | --- | --- | --- |
| Never | Rarely | Sometimes | Always |
| 1 | 2 | 3 | 4 |

| **Domain II- Lack of Emotions and Sensitivity.** |
| --- |

| 1. I fail to perceive patient’s situation and feelings. | | | |
| --- | --- | --- | --- |
| Never | Rarely | Sometimes | Always |
| 1 | 2 | 3 | 4 |

| 1. I don’t take extra mile to help the patient. | | | |
| --- | --- | --- | --- |
| Never | Rarely | Sometimes | Always |
| 1 | 2 | 3 | 4 |

| 1. I feel disconnected to my patients and co-workers. | | | |
| --- | --- | --- | --- |
| Never | Rarely | Sometimes | Always |
| 1 | 2 | 3 | 4 |

| 1. I lack curiosity in learning new things. | | | |
| --- | --- | --- | --- |
| Never | Rarely | Sometimes | Always |
| 1 | 2 | 3 | 4 |

| 1. I do not communicate my understandings to the client. | | | |
| --- | --- | --- | --- |
| Never | Rarely | Sometimes | Always |
| 1 | 2 | 3 | 4 |

| **Domain III- Lack of Interest.** |
| --- |

| 1. I do not feel interested while handling new cases. | | | |
| --- | --- | --- | --- |
| Never | Rarely | Sometimes | Always |
| 1 | 2 | 3 | 4 |

| 1. I avoid spending time with patients. | | | |
| --- | --- | --- | --- |
| Never | Rarely | Sometimes | Always |
| 1 | 2 | 3 | 4 |

| 1. I feel boring while working with some people I helped. | | | |
| --- | --- | --- | --- |
| Never | Rarely | Sometimes | Always |
| 1 | 2 | 3 | 4 |

| 1. I feel depressed of caring critically ill patients on regular routine. | | | |
| --- | --- | --- | --- |
| Never | Rarely | Sometimes | Always |
| 1 | 2 | 3 | 4 |

| 1. I feel exhausted when I get up in the morning and have to face another day on job. | | | |
| --- | --- | --- | --- |
| Never | Rarely | Sometimes | Always |
| 1 | 2 | 3 | 4 |

| 1. I think that I am experiencing a trauma of a person I care for. | | | |
| --- | --- | --- | --- |
| Never | Rarely | Sometimes | Always |
| 1 | 2 | 3 | 4 |

| **Domain IV- Intrusive thoughts.** |
| --- |

| 1. I feel like pre-occupied with person I helped. | | | |
| --- | --- | --- | --- |
| Never | Rarely | Sometimes | Always |
| 1 | 2 | 3 | 4 |

| 1. I feel like being in danger while caring traumatised patients. | | | |
| --- | --- | --- | --- |
| Never | Rarely | Sometimes | Always |
| 1 | 2 | 3 | 4 |

| 1. I get intrusive thoughts of terrible experiences of caring others. | | | |
| --- | --- | --- | --- |
| Never | Rarely | Sometimes | Always |
| 1 | 2 | 3 | 4 |

| 1. I experience flashbacks of those critically ill persons I helped | | | |
| --- | --- | --- | --- |
| Never | Rarely | Sometimes | Always |
| 1 | 2 | 3 | 4 |

| **Domain V- Hopelessness** |
| --- |

| 1. I feel that I am not successful in my role as a helper or caregiver | | | |
| --- | --- | --- | --- |
| Never | Rarely | Sometimes | Always |
| 1 | 2 | 3 | 4 |

| 1. I feel very weak as a result of my job as a helper. | | | |
| --- | --- | --- | --- |
| Never | Rarely | Sometimes | Always |
| 1 | 2 | 3 | 4 |

| 1. I do not view my failings as part of the human condition. | | | |
| --- | --- | --- | --- |
| Never | Rarely | Sometimes | Always |
| 1 | 2 | 3 | 4 |

| 1. I find it difficult to recover easily after facing daily troubling events. | | | |
| --- | --- | --- | --- |
| Never | Rarely | Sometimes | Always |
| 1 | 2 | 3 | 4 |

| **Domain VI- Worthlessness.** |
| --- |

| 1. I have a sense of worthlessness associated with my work. | | | |
| --- | --- | --- | --- |
| Never | Rarely | Sometimes | Always |
| 1 | 2 | 3 | 4 |

| 1. I really do not care about my patients. | | | |
| --- | --- | --- | --- |
| Never | Rarely | Sometimes | Always |
| 1 | 2 | 3 | 4 |

| 1. I do not feel sincere while caring for patients. | | | |
| --- | --- | --- | --- |
| Never | Rarely | Sometimes | Always |
| 1 | 2 | 3 | 4 |

| **Domain VII- Overwhelming** |
| --- |

| 1. I feel overwhelmed while caring for critically ill patients. | | | |
| --- | --- | --- | --- |
| Never | Rarely | Sometimes | Always |
| 1 | 2 | 3 | 4 |

| 1. I am deeply affected by my patients’ stress. | | | |
| --- | --- | --- | --- |
| Never | Rarely | Sometimes | Always |
| 1 | 2 | 3 | 4 |

| 1. I experience frightening dreams at night about the people I helped during the day. | | | |
| --- | --- | --- | --- |
| Never | Rarely | Sometimes | Always |
| 1 | 2 | 3 | 4 |

| **Domain VIII- Compassion.** |
| --- |

| 1. I feel drained rather than energized after caring for others. | | | |
| --- | --- | --- | --- |
| Never | Rarely | Sometimes | Always |
| 1 | 2 | 3 | 4 |

| 1. I do not feel proud of what I do to help others. | | | |
| --- | --- | --- | --- |
| Never | Rarely | Sometimes | Always |
| 1 | 2 | 3 | 4 |

| 1. I do not feel a sense of compassion in the work I do. | | | |
| --- | --- | --- | --- |
| Never | Rarely | Sometimes | Always |
| 1 | 2 | 3 | 4 |

| **Domain IX- Competency** |
| --- |

| 1. I find myself incompetent in my work | | | |
| --- | --- | --- | --- |
| Never | Rarely | Sometimes | Always |
| 1 | 2 | 3 | 4 |

| **Domain X- Productivity.** |
| --- |

| 1. My productivity at work has been reduced | | | |
| --- | --- | --- | --- |
| Never | Rarely | Sometimes | Always |
| 1 | 2 | 3 | 4 |
